# Soybean Meal-Induced Intestinal Inflammation in Zebrafish Is T Cell-Dependent and Has a Th17 Cytokine Profile

**DOI:** 10.3389/fimmu.2019.00610

**Published:** 2019-04-02

**Authors:** Maximo Coronado, Camila J. Solis, Pedro P. Hernandez, Carmen G. Feijóo

**Affiliations:** ^1^Departamento de Ciencias Biologicas, Facultad de Ciencias de la Vida, Universidad Andres Bello, Santiago, Chile; ^2^Millennium Nucleus in the Biology of Intestinal Microbiota, Santiago, Chile; ^3^Escuela de Tecnología Médica, Facultad de Ciencias de la Salud, Universidad San Sebastian, Santiago, Chile; ^4^Macrophages and Development of Immunity, Institute Pasteur, Paris, France

**Keywords:** intestinal inflammation, zebrafish, innate immune, adaptive immunity, Th17 T cells, macrophage, lymphocyte

## Abstract

Currently, inflammatory bowel disease (IBD) is a serious public health problem on the rise worldwide. In this work, we utilized the zebrafish to introduce a new model of intestinal inflammation triggered by food intake. Taking advantage of the translucency of the larvae and the availability of transgenic zebrafish lines with fluorescently labeled macrophages, neutrophils, or lymphocytes, we studied the behavior of these cell types *in vivo* during the course of inflammation. We established two feeding strategies, the first using fish that were not previously exposed to food (naïve strategy) and the second in which fish were initially exposed to normal food (developed strategy). In both strategies, we analyzed the effect of subsequent intake of a control or a soybean meal diet. Our results showed increased numbers of innate immune cells in the gut in both the naïve or developed protocols. Likewise, macrophages underwent drastic morphological changes after feeding, switching from a small and rounded contour to a larger and dendritic shape. Lymphocytes colonized the intestine as early as 5 days post fertilization and increased in numbers during the inflammatory process. Gene expression analysis indicated that lymphocytes present in the intestine correspond to T helper cells. Interestingly, control diet only induced a regulatory T cell profile in the developed model. On the contrary, soybean meal diet induced a Th17 response both in naïve and developed model. In addition, when feeding was performed in *rag1*-deficient fish, intestinal inflammation was not induced indicating that inflammation induced by soybean meal is T cell-dependent.

## Introduction

Intestinal inflammation (or enteritis) is a feature of several chronic pathologies such as inflammatory bowel disease (IBD) in humans as well as of similar pathologies in fish (1–3). One of the earliest signs of intestinal inflammation is the infiltration of neutrophils into the gut mucosa and the epithelial layer, in addition to polarization of macrophages and dendritic cells toward an inflammatory phenotype (4–6). Enteritis is also characterized by a drastic increase of natural killer cells in the gut as well as activation of mast cells (7). Each of these cell types secretes specific cytokines that trigger several pathways characteristic of enteritis (7). On the other hand, CD4^+^ T helper (Th) cells are critical for proper immune cell homeostasis and host defense but are also major contributors to the pathology of autoimmune and inflammatory diseases (8). Depending on the cytokine milieu, different Th subsets can be induced, such as Th1, Th2, Th17, Th22, and Th9, each with specific functional outcomes (9). Likewise, regulatory T cells (Tregs) are essential for the development of tolerance to self and non-self antigens. Activation of Tregs inhibits the inflammatory response to commensal bacteria and is central for mucosal tolerance (10). Conversely, functional defects in Tregs underlie infectious, autoimmune and chronic inflammatory conditions, including IBD (11, 12).

Several models for IBD have been developed in the mouse model. Based on how inflammation is brought about, they can be categorized into four groups: chemical models, genetically engineered models, cell transfer models, and congenic models. Although each of them covers a specific aspect of this pathological condition, none of them encompasses the spontaneous and fluctuating nature of the human disease (13). Thus, in order to extrapolate the experimental findings from mouse studies toward the improvement of knowledge and therapy in IBD pathogenesis in humans, it is necessary to understand the specific advantages and limitations of each model (14, 15). One of the key limitations shared between all models is the difficulty to follow cell behavior and gene function *in vivo*. To overcome this situation, we propose the use of the zebrafish (*Danio rerio*) for studying the contribution of different immune cell types to intestinal inflammation *in vivo*. This teleost fish constitutes a unique tool that allows to combine live imaging of specific fluorescently-labeled cell types with molecular strategies to manipulate gene function to monitor the course of an inflammatory process in real-time and at the whole organism level (16–19). Moreover, the anatomy and architecture of the zebrafish intestine closely resembles the one of mammals, and all main immune cell lineages have been described in this vertebrate model (20–22). Importantly, most chemical-induced intestinal inflammation models used in mice have also been used in zebrafish with similar results (23–25). In order to obtain a more physiological intestinal inflammation model, we established a novel strategy in zebrafish larvae based on the intake of a soybean meal-based diet (26). Using this approach, we have reported that, as early as 2 days after feeding, the number of neutrophils increased in the gut, as well as the mRNA levels of several proinflammatory cytokines such as *il1b* and *cxcl8*. Conversely, no changes in intestinal architecture were detected, suggestive of an early stage in the inflammatory process (26). In this new study, we have compared the behavior of innate cells such as neutrophils, macrophages, and mast cells, in addition to T cells, between two conditions: naïve intestine (not previously exposed to food) and developed intestine (already exposed to regular food). Our findings show that innate immune responses were similarly triggered after fish maintained under both conditions are afterwards exposed to a control or inflammatory diet. T cells, in contrast, responded differently. In the case of naïve intestines, an inflammatory process with increased numbers of helper T cells was induced under both control and inflammatory diet (soybean meal-based diet), the latter with a Th17 profile. Conversely, in developed intestines, the control diet triggered a tolerogenic response with abundant Treg cells, and the inflammatory diet a Th17 profile with decreased presence of Treg cells. When *rag1*^−/−^ fish were fed with inflammatory diet, no increase in neutrophils, or lymphocytes was observed, indicating that T cells are needed to trigger immune responses to soybean meal. These results demonstrate, for the first time, functional adaptive immune response in zebrafish as early as 5 days post-fertilization. They also reveal an evolutionarily conserved response between zebrafish and mammals, supporting the suitability of the zebrafish model to study intestinal inflammation with biomedical purposes.

## Materials and Methods

### Zebrafish Strains and Maintenance

Zebrafish were maintained and bred in our facility according to standard protocols (27). The following strains of fish were used in this study: Tab5, Tg(lck:lck-eGFP) (28), Tg(lysC:DsRed) (29), Tg(mpeg1:Dendra2) (30), and *rag1*^−/−^ mutants (31). All embryos were collected by natural spawning and maintained at 28°C in E3 medium (5 mM NaCl, 0.17 mM KCl, 0.33 mM CaCl2, 0.33 mM MgSO4, with methylene blue, equilibrated to pH 7.0) in petri dishes (32). Embryonic and larval ages are expressed in hours post fertilization (hpf) or days post fertilization (dpf).

### Feeding Strategies

Two feeding strategies were used; the naïve feeding strategy and the developed feeding strategy (Supplementary Figure 1A). The naïve feeding protocol comprised larval feeding from 5 dpf (T0n) to 9 dpf (T4) with a control diet (fishmeal-based diet) or an inflammatory diet (soybean meal-based diet). Diets were prepared as described previously (26). As controls we included non-fed larvae from 9 dpf (T4n). The developed feeding protocol consisted of two parts; the maintenance feeding regime where larvae were fed from 5 to 17 dpf with regular food (gemma micron 300) and the experimental feeding phase where larvae were fed from 18 to 25 dpf with control or inflammatory diet. In all cases larvae were fed *ad libitum* and maintained in 100 ml fish water in a 200 ml tank at 28°C. The last feeding was performed 19 h before processed for the different analyses performed, qPCR, immunohistochemistry or imaging.

### Immunohistochemistry

**Figure 1:**
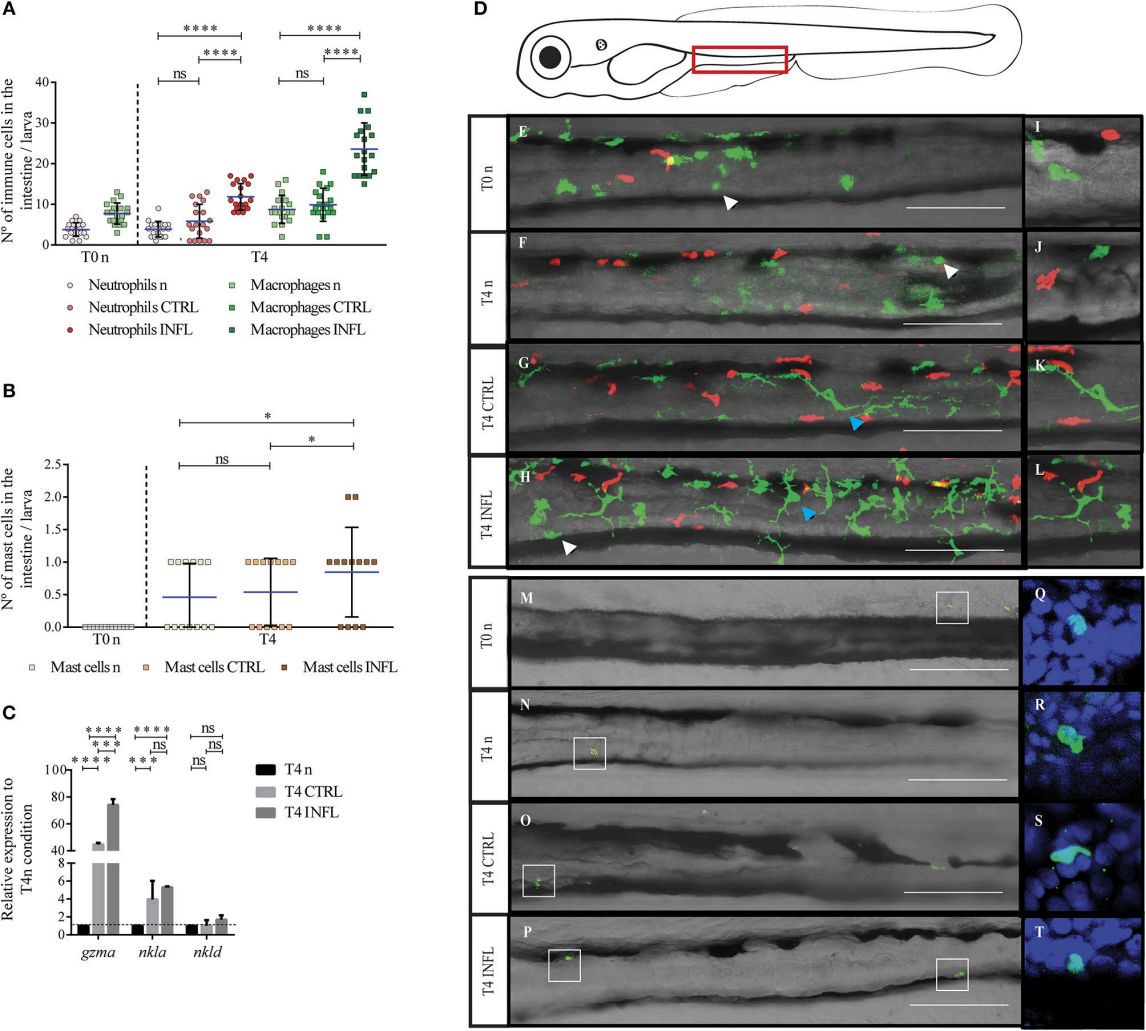
Neutrophils, macrophages, and mast cells increase their presence in the gut during the inflammatory process triggered in the naïve feeding model. The number of neutrophils/macrophages **(A)**, and mast cells **(B)** was quantified under different conditions (n, naïve; CTRL, control; INFL, inflammation) and time points (T0: 5 dpf; T4: 9 dpf). **(C)** Relative mRNA levels of *gzma, nkla*, and *nkld* were analyzed. Data were normalized against *rpl13a* and compared to T4 naïve condition (dotted line). **(D)** Scheme of a lateral view of a 9 dpf larva with the zone of the intestine used to quantify delimited by the red rectangle. **(E–H)** Lateral view of the intestine of a Tg(lysC:DsRed)xTg(mpeg1:Dendra2) larva showing fluorescently labeled neutrophils (red) and macrophages (green). **(M–P)** Lateral view of the intestine showing mast cells labeled by anti-tryptase immunohistochemistry. Zoom of macrophages and neutrophils **(I–L)** and mast cells **(Q–T)**. ^*^p < 0.05; ^***^p < 0.001; ^****^p < 0.0001. Scale bar 200μm. Experiments were done at least in three biological replicates with 20 individuals per condition.

Immunohistochemistry was performed essentially as previously described (33). Briefly, larvae were fixed for 1 h in 4% paraformaldehyde in phosphate-buffered saline (PBS), then rinsed in PBS + 1% Tween 20 (PBS-Tween), dehydrated in 100% methanol and stored at −20°C until use. The following antibodies were used: rabbit anti-GFP (Invitrogen cat: A11122), anti-MPO (Genetext cat: GTX128379) and anti-tryptase (Abcam cat: ab2378). Quantification of neutrophils, mast and lymphoid cells in the intestine was performed in whole-mount larvae; for this purpose, a region in the midgut was defined (red rectangle in Figure 1). Thirty larvae per condition were analyzed in three different experiments.

### Proliferation Assay

Tg(lck:lck-eGFP) larvae were exposed to the nucleotide analog 5-ethynyl-2′-deoxyuridine (EdU) as was previously described (34) with some modifications. Larvae were immersed in 100 μg/ml EdU (Invitrogen) with 0.5% DMSO for 16 h and fixed 1 h at RT with 4% PFA in PBS. Larvae were then processed using the Click-iT EdU Imaging Kit (Invitrogen) according to manufacturer's instructions.

### Histological Section

Twenty-five dpf larvae were fixed in Bouin's solution for 3 h at RT, washed twice in water and mounted in 3% low melting agarose (Cleaver) for paraffin embedding and sectioning as previously described (26). Sections of 5 μm were obtained and stained with hematoxylin & eosin (Merck.) The quantification of the number of intestinal folds was analyzed as previously described by Hedrera et al. (26).

### Confocal Microscopy

For time-lapse and photography, larvae were anesthetized with tricaine 4% (Fluka, cat: 1001011075), mounted in low-melting agarose 1% in E3 and registered under a Leica TCS Sp8 microscope, with Leica application suite X (LAS X software version 3.1.5) and using an optical zoom of 40 and 60x. The images were analyzed with Image J 1.44, representing at least 70% of each population.

### Intestine Dissection and RT-qPCR

Intestines were dissected at different times points according to Supplementary Figure 1A. Dissections were performed with entomological pins in RNAasa free conditions. One hundred intestines per condition were directly stored in TRIzol reagent (Invitrogen, cat: 15596-026) for total RNA extraction. Extraction was performed according to the manufacturer's instructions. Synthesis of cDNA was performed with 1 μg RNA and SuperScript II RT (Invitrogen, cat: 100004925), according to manufacturer's instructions and using oligo-dT primers. Real-time PCR was performed following the methodology described previously (35). The mean Ct values from each sample were normalized against the mean Ct value of a reference gene (*rpl13a*, housekeeping gene). The genes analyzed are detailed in Table 1. All experiments were performed on at least three biological replicates.

**Table 1:** Sequences of primers used in qPCR.

**Gene**	**Forward primer**	**Reverse primer**	**Accession number**
*rpl13a*	TCTGGAGGACTGTAAGAGGTATGC	AGACGCACAATCTTGAGAGCAG	NM_212784
*il17a/f1*	CATTCGGTGCTGAGGGGG	AGCCGGTATGAATGATCTGC	NM_001020787
*il17a/f3*	AAGATGTTCTGGTGTGAAGAAGTG	ACCCAAGCTGTCTTTCTTTGAC	NM_001020790
*il22*	GATGACTGATACAGCACGAAA	CATTGATGCAGCAGGAACCT	NM_001020792
*rorca*	TCTTTTCCTATCCAACCTCTCTACA	GAGTGGTCTCTTTATGTGAGCGTA	XM_001344013
*gzma*	GATTTTTGGCTGAGAGGACGG	ACGTCAGAGCAAACTGTCACT	XM_001335130
*nkla*	GATGACGAATGACGGAGTAAAC	TCTCATTCACAGCCCGGT	NM_001311794
*nkld*	TGTGATCAGATCGGGTTCCT	AGCACAGATGGTTCTGGCAT	NM_212741
*il10*	TCACGTCATGAACGAGATCC	CCTCTTGCATTTCACCATATCC	NM_001020785
*foxp3a*	CTCGGCTCATCTCGCAATCA	CGGTGTCCACAACCCAATCA	NM_001329567
*ccr9a*	TGCACCATGGTCTACTGGAA	ATAACCCGAAGTGCCTTGTG	NM_001244716
*ccl25*	ACATCCCAGCCATTGTCTTC	GCTGAAATGAGCCCTCGTAG	XM_002660965
*lck*	GCCGAAGAAGATCTCGATGGT	TCCCCATGTTTACGTATTTTGTCG	NM_001001596
*trac*	TCGTTTTCAATGTGCTGGTG	GATGATCTGGAATGGGATGC	NM_001199372
*cd4.1*	AAGAGTTGAGAAAGCTCCAGTG	CTGGTCTTGCGTCGTCTGTA	NM_001135096
*mhczea*	GGCTGTTTTTGCCGCTCTG	GTGGACAGGTCTGGATAAAG	NM_001089550
*mhc2ab*	CTCTGTGGGGAAGTTTGTG	CCAGATCCGAGCATTATGTC	NM_131476
*hamp*	GCCGTTCCCTTCATACAGCA	CCTGAACAGAACCAGAGGGTC	NM_205583
*leap2*	TGTGGGTACTAAACCACACGG	GCCCATCCTGCATATTCCTGT	NM_001128777
*trim33*	GAACCCGAACTCCAGAGCAA	AGCATTAGTAGCACCGCCTC	NM_001002871

### Statistics

Statistical analysis for quantification of cell numbers was performed using unpaired *t*-test or a non-parametric test, the Kruskall–Walls one-way ANOVA test. RT-qPCR analyses were performed using Kruskall–Walls one-way ANOVA test. All analyses were made using Prim 6 (GraphPad Software). The significance level was set to *P* < 0.05.

## Results

To analyze mucosal immune responses in intestines of fish exposed for first time to food, we used a well-established inflammation model (26), which consists of 4 days of feeding with soybean meal-based diet, from 5 to 9 dpf (hereafter, naïve model) (Supplementary Figure 1A). To address if inflammation induced by this model remits after treatment, we carried out the naïve model and after that, fed larvae with control diet during 4 more days. We then quantified the number of neutrophils present in the intestine. As expected, larvae fed the inflammatory diet had significantly more neutrophils than larvae fed the control diet at 4 days of treatment (9 dpf, T4); 9.5 and 4.3, respectively. Then, 4 days after feeding with the inflammatory diet (T4+4) the number of neutrophils in the intestine of control and inflamed larvae was similar: 4.7 and 5.9, respectively (Supplementary Figure 1B). In order to provide a model more comparable to mammalian intestinal inflammatory models, in which the intestine has been exposed to food for a considerable period of time, we established a new intestinal inflammation model, hereafter “developed model.” This new strategy comprised a first step of 12 days of feeding with regular food (from 5 to 17 dpf), followed by the second step of 8 days of experimental feeding (from 18 to 25 dpf) (Supplementary Figure 1A). In this approach, the gut is first exposed to a regular diet for a few days, allowing the differentiation and functionality of intestinal cell types as well as the colonization of commensal microbes (36). To determine if the inflammation triggered in the developed model is sufficient to induce changes in the morphology of the intestine, we analyzed histological cross sections of the midgut of larvae fed with control and experimental diets (Supplementary Figure 1C). We found that the number of folds present in the intestine of larvae fed the inflammatory diet was significantly lower than the number of folds present in control larvae, 9.12 and 9.78, respectively, (Supplementary Figure 1D), indicating that after 8 days of feeding, the inflammation caused by the developed model is detectable at a morphological level.

### Neutrophils, Macrophages, Mast Cells, and Natural Killer Cells Respond Similarly to Innocuous and Harmful Food During Naïve Feeding Model

To analyze innate immune responses triggered upon inflammation in the naïve model, we monitored neutrophil, macrophage, and mast cell behavior. We took advantage of the double transgenic line Tg(lysC:DsRed)xTg(mpeg1:Dendra2), in which neutrophils and macrophages are fluorescently labeled in red and green, respectively. To evaluate mast cells, we performed immunofluorescence with an anti-tryptase antibody, which has been shown to label specifically mast cells (37). Using confocal microscopy, we determined cell morphology and the number of infiltrated cells in the intestine at two different time points: T0n (5 dpf, before feeding) and T4 (9 dpf, after feeding) (Figure 1). Also, we included a control with larvae without feeding at 9 dpf (T4n). We found that in both unfed conditions, T0n and T4n, there were 4 to 5 neutrophils in the intestine (Figure 1A). Later, at T4, we found a similar number of neutrophils. Conversely, inflammatory diet induced an increase to an average of 12 neutrophils per intestine (Figure 1A). In the case of macrophages, both naïve conditions (T0n and T4n) displayed a mean of 9 cells in the intestine. Similarly, to neutrophils, the number of macrophages at T4 only increased in larvae fed with the inflammatory diet, with 23 cells per intestine (Figure 1A). Remarkably, the number of neutrophils and macrophages did not increase due to larval development, T0 vs. T4n, suggesting that the increase of both types of immune cells is due to the inflammatory response. Finally, we did not observe mast cells in intestine at T0 and T4n and after feeding, in T4, larvae fed with control and inflammatory diet displayed a mean of 1 mast cell per intestine (Figure 1B).

To further characterize the participation of other innate immune cells during the naïve model of inflammation, we analyzed the natural killer cell markers Granzyme A (*gzma*), NK lysin-a (*nkla*), and NK lysin-d (*nkld*) by RT-qPCR (38). We found increased expression of *gzma* and *nkla* in both control and inflammatory diet compared to the naïve situation (T4n), suggesting the participation of NK cells in the response triggered by both the inflammatory and the control diet (Figure 1C).

### Macrophage Morphology Changes After the Intestine Is Exposed to Food

Cell morphology can change when cell function changes, we thus evaluated if the inflammatory diet induces alterations in the morphology of myeloid cells. To this end, we analyzed the shape of neutrophils, macrophages and mast cells by confocal microscopy before (T0n, T4n) and after feeding (T4). At T0n and T4n, neutrophils displayed a rounded shape that switched to an elongated morphology (Figures 1E,F,I,J, red cells), probably due to increased motility. Later, at T4, neutrophil morphology was similar to that observed before feeding, both under control and inflammatory conditions (Figures 1G,H,K,L, red cells). Likewise, mast cells displayed a rounded shape at T0 and T4n, which remained unaltered among the different conditions at T4 (Figures 1M–P,Q–T). Macrophages, on the other hand, displayed a rounded shape at T0 and T4n (Figures 1E,F,I,J, green cells) and a size similar to neutrophils. Conversely, after food intake, either under the control or the inflammatory diet, morphology drastically changed, showing cells with several long protrusions and increased size compared to the naïve situation (Figures 1G,H,K,L). Thus, these results suggest that the activation and/or function of macrophages change upon feeding, regardless if the food is innocuous or inflammatory.

### Neutrophils, Macrophages, Mast Cells, and Natural Killer Cells Respond Similarly to Food Antigens, Either Innocuous or Inflammatory Upon the Developed Feeding Model

**Figure 2:**
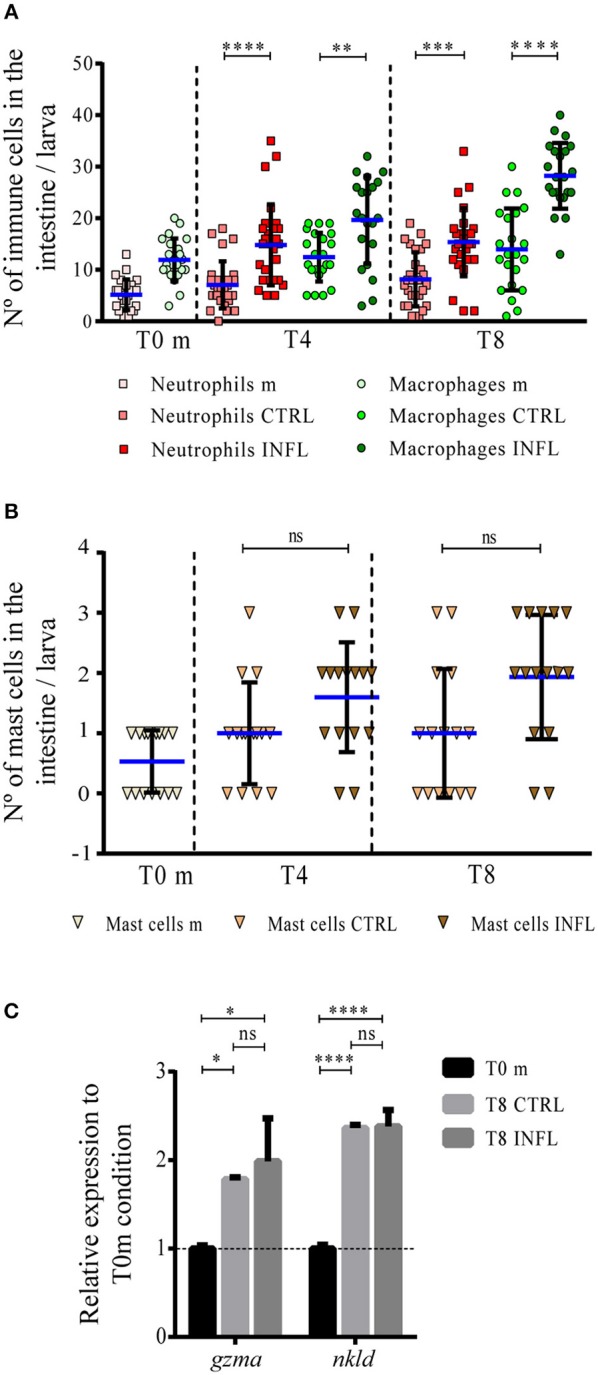
Neutrophils and macrophages increase in number in the gut during the inflammation process triggered in the developed feeding model. The number of neutrophils/macrophages **(A)**, and mast cells **(B)** was quantified under the different conditions (m, maintenance; CTRL, control; INFL, inflammation) and time points (T0m: 18 dpf; T4: 21 dpf; T8: 25 dpf). **(C)** Relative mRNA levels of *gzma* and *nkld* were analyzed. Data were normalized against *rpl13a* and compared to T0 maintenance condition (dotted line). ^**^p < 0.01; ^***^p < 0.001; ^****^p < 0.0001. Experiments were done at least in three biological replicates with 20 individuals per condition.

In the case of the developed feeding model, the number of neutrophils, macrophages and mast cells in the gut at T0 was similar to that observed at the same time-point in the naïve feeding protocol, with 5, 9, and 1 cells, respectively, (Figures 2A,B). During the inflammatory condition, both neutrophils and macrophages increased in the gut from T0 to T4, with neutrophils remaining constant up to T8 and macrophages maintaining their increment (Figure 2A). Meanwhile, the number of mast cells did not vary during the entire period, as observed in the naïve feeding model (Figure 2B). Finally, mRNA levels of NK cell markers increased under control and inflammatory conditions, compared to the regular maintenance condition (Figure 2C). In summary, these results indicate that if the intestine is exposed to antigens for first time or after continuous exposure to food, the response exerted by innate immune cells is similar.

### Lymphoid Cells Colonize the Gut at Early Larval Stages and Are Able to Respond to Inflammatory Stimuli

**Figure 3:**
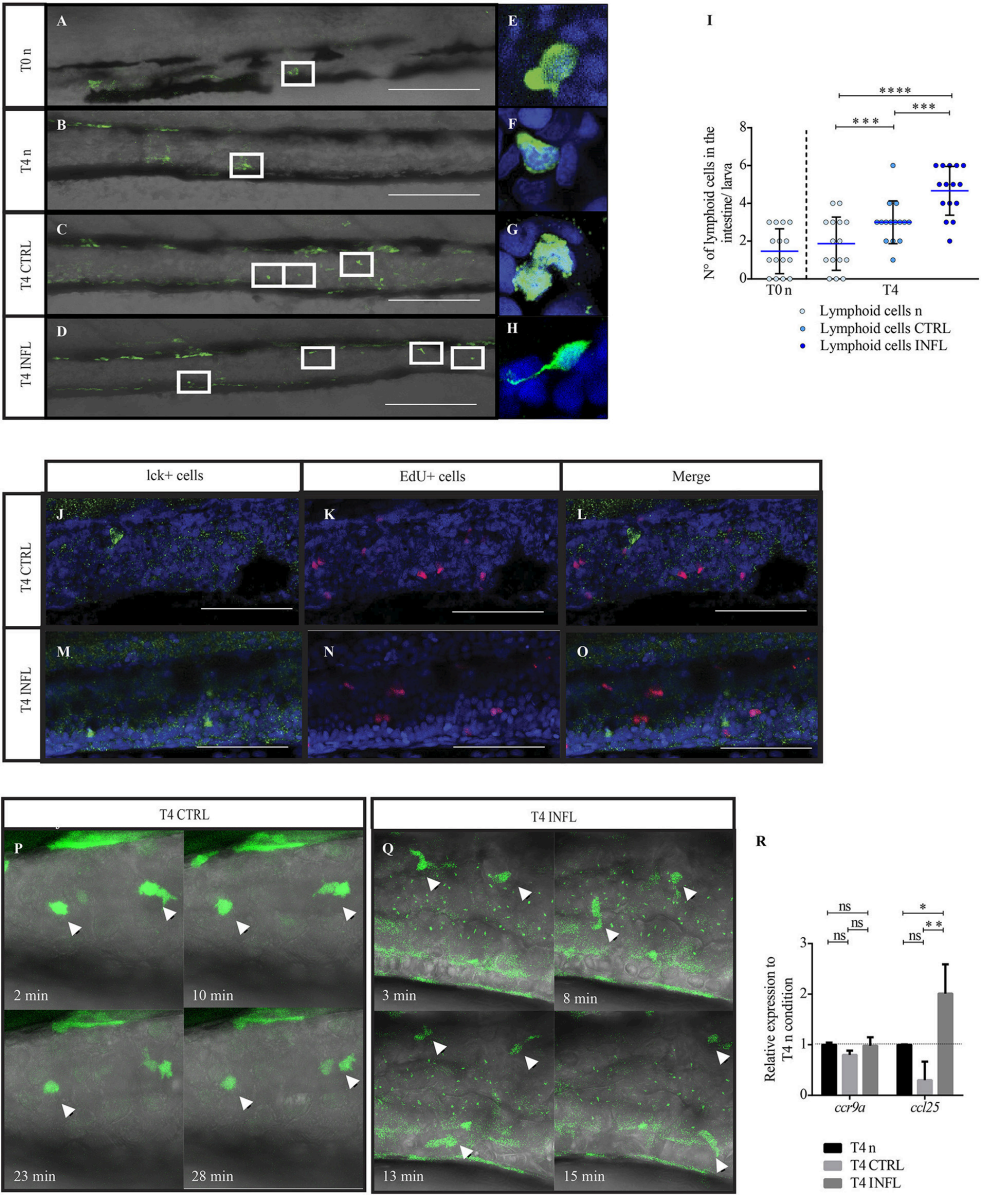
Lymphoid cells are present in the intestine as early as 5 dpf and are able to respond to food antigens. **(A–D)** Lateral view of the intestine from a Tg(lck:lck-eGFP) larva showing fluorescently labelled lymphoid cells (green). **(E–H)** Higher magnification of lymphocytes under the different conditions. **(I)** Quantification of lymphoid cells under the different conditions (n: naive; CTRL: control; INFL: inflammation) and time points (T0: 5 dpf; T4: 9 dpf). **(J–O)** T cells in control **(J)** or inflamed **(M)** intestine do not colocalize with EdU+ cells **(K,L,N,O)**. **(P)** Example of migration of T cells (arrowhead) in a Tg(lck:lck-eGFP) larvae fed with control diet (derived from Supplementary Movie 1). **(Q)** Example of the migration of T cells (arrowheads) in a Tg(lck:lck-eGFP) larva fed with inflammatory diet (derived from Supplementary Movie 2). **(R)** Relative mRNA levels of ccl25 and ccr9a were analyzed in the intestine of control and inflamed larvae. Data was normalized against rpl13a and compared to naïve or maintenance condition (dotted line). ^*^p < 0.05; ^**^p < 0.01; ^***^p < 0.001; ^****^p > 0.0001. Scale bar for **A–D** 200 μm and **J–O** 100 μm. Experiments were done at least in three biological replicates with 20 individuals per condition.

Currently available literature indicates that zebrafish develop a functional adaptive immune system only after 3 weeks of development (18). Since we observed that the inflammatory diet triggered a strong myeloid cell response at 9 dpf, we decided to investigate if lymphoid cells are present at this developmental stage and if they are able to respond to the different food antigens. To this end, we used the Tg(lck:lck-eGFP) transgenic line with fluorescently labeled lymphoid cells (28). At 5 dpf, before feeding, we found few lymphoid cells (1 or 2) in the gut (Figures 3A,I). At this developmental stage, the intestine has a freshly formed lumen (39). Later, at 9 dpf (T4n), the number of lymphoid cells present in the gut of naïve larvae remained unchanged (Figures 3B,I). On the other hand, the intake of both diets, control and inflammatory, triggered an increase of lymphoid cells in the intestine, 3 and 4 cells, respectively, (Figures 3C,D,I). Regarding morphology, we compared lymphoid cell shape between all conditions using confocal microscopy. We found that in both naïve conditions, T0 and T4n, and after feeding either control or inflammatory diet, lck^+^ cells displayed a rounded shape with a lower proportion of cytoplasm and a prominent nucleus (Figures 3E–H). Interestingly, in the inflammatory condition, we found few lck^+^ cells with long protrusions (Figure 3H).

To determine if the increase in lck^+^ cells observed in larvae fed the inflammatory diet is due to new cells recruitment or due to proliferation, we incubated larvae with thymidine nucleoside (EdU) for 16 h and then immunodetected it. We did not observe any colocalization between lck^+^ cells and EdU^+^ cells (Figures 3 J–O). To corroborate this results we performed immunofluorescence against phosphorylated Histone 3 (H3P) in Tg(lck:lck-eGFP). As in the EdU assay, we did not find any colocalization between H3P^+^ cells and lck^+^ cells (data not shown).

To analyze the behavior of lymphoid cells during the control and the inflammatory condition, we performed time-lapse analysis (Figures 3P,Q). We found that GFP^+^ cells did not display motility at steady-state, they only moved their protrusions but without displacement (Figure 3P, Supplementary Movie 1). Interestingly, during inflammation, GFP^+^ cells showed substantial and continuous motility along the intestine (Figure 3Q, Supplementary Movie 2). This behavioral change could be an indicator of presence of T cells in an activated state. Finally, and to determine if lymphoid cell recruitment to the intestine is regulated by similar signaling pathways than in mammals, we quantified mRNA levels of the *ccr9a* receptor and its ligand *ccl25*. Our results showed that in both feeding conditions, *ccl25* mRNA increased upon inflammation (Figure 3R), suggesting evolutionary conservation of signaling pathways regulating T cell recruitment to the intestine.

### Lymphoid Cells Present in the Intestine Are Helper T Cells With a Th17 Transcriptional Profile

**Figure 4:**
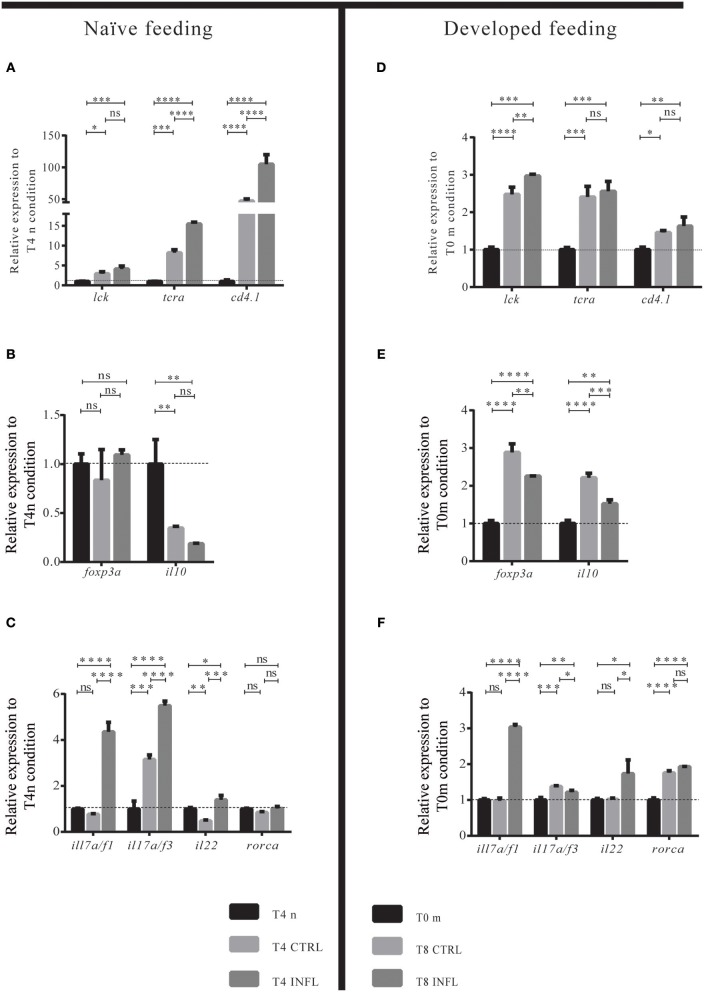
Intestinal lymphoid cells are CD4^+^ T cells with a Th17-like transcriptional profile. **(A–F)** Relative transcriptional level of lymphoid-associated genes was analyzed. Data was normalized against *rpl13a* and compared to the naïve condition (T0n; dotted line) or to the maintenance condition, T0m. For each condition, 100 guts were analyzed, and three biological replicates were made. ^*^p < 0.05; ^**^p < 0.01; ^***^p < 0.001; ^****^p > 0.0001.

To determine if the lymphoid cells present in the intestine were T cells and if they have a specific Th profile, we evaluated general T cell markers (*lck; trac; cd4.1; cd8*), as well as Treg (*foxp3a* and *il10*), and Th17 (*il17a/f1; il17a/f3; il22; rorca*) markers. We compared the expression levels of all these genes between naïve (or maintenance) condition vs. control or inflammatory diet. In the case of the naïve feeding strategy, we observed an increase in the mRNA levels of *lck* and *trac* in the control and inflammatory conditions compared with the naïve situation. In contrast, there was no difference in the mRNA levels between the control with the inflammatory condition (Figure 4A). Also, we evaluated *cd4-1* expression, which strongly increased in the control and inflammatory conditions compared to the naïve situation (Figure 4A). It is important to highlight that, although the mRNA levels of *cd8* were analyzed, expression was not detected (data not shown). These results suggest that lymphoid cells increase in fed larvae, regardless of the type of food and that at least part of them are helper T cells. Similarly, in the developed feeding model, *lck, trac*, and *cd4.1* expression increased in control and inflammatory conditions at T8 when compared to T0m (Figure 4D). In the naïve model, we also evaluated if *mhc1zea, mhc2ab, hamp, leap2, and trim33* genes changed their mRNA level between control and inflammatory conditions. We found an increase in *mhc1zea* and *mhc2ab* genes in larvae fed the inflammatory diet. On the contrary, none of the antimicrobial peptide markers genes, *hamp, leap2, and trim33*, altered their transcription level (Supplementary Figure 2).

To determine the type of Th profile acquired by helper T cells after intake of the control or inflammatory diet, we quantified the mRNA levels of the Th17 markers *il17a/f1, il17a/f3, il22*, and *rorca*, and the Treg markers *foxp3a* and *il10*. After the naïve feeding model, larvae displayed increased numbers of intestinal T cells, however, they did not show a defined Treg or Th17 profile since only *il17a/f1* was increased (Figures 4B,C). In contrast, in the developed feeding model, T cells displayed a Treg profile with an increase in the mRNA levels of *foxp3a* and *il10* (Figure 4E). In the case of the inflammatory diet, the mRNA levels of Th17 gene markers were increased while Treg markers decreased compared to control conditions, both in the naïve and developed feeding models (Figures 4B,C,E,F). In summary, these results show that in a naïve intestine, helper T cells do not polarize to a specific Th profile in response to innocuous and noxious food antigens, responding similarly in both cases. Conversely, in an intestine already exposed to food, helper T cells selectively detect innocuous or noxious antigens, triggering a tolerogenic or inflammatory response, respectively.

### Soybean Meal Based Inflammatory Diet Triggered Intestinal Inflammation in a T Cell-Dependent Manner

**Figure 5:**
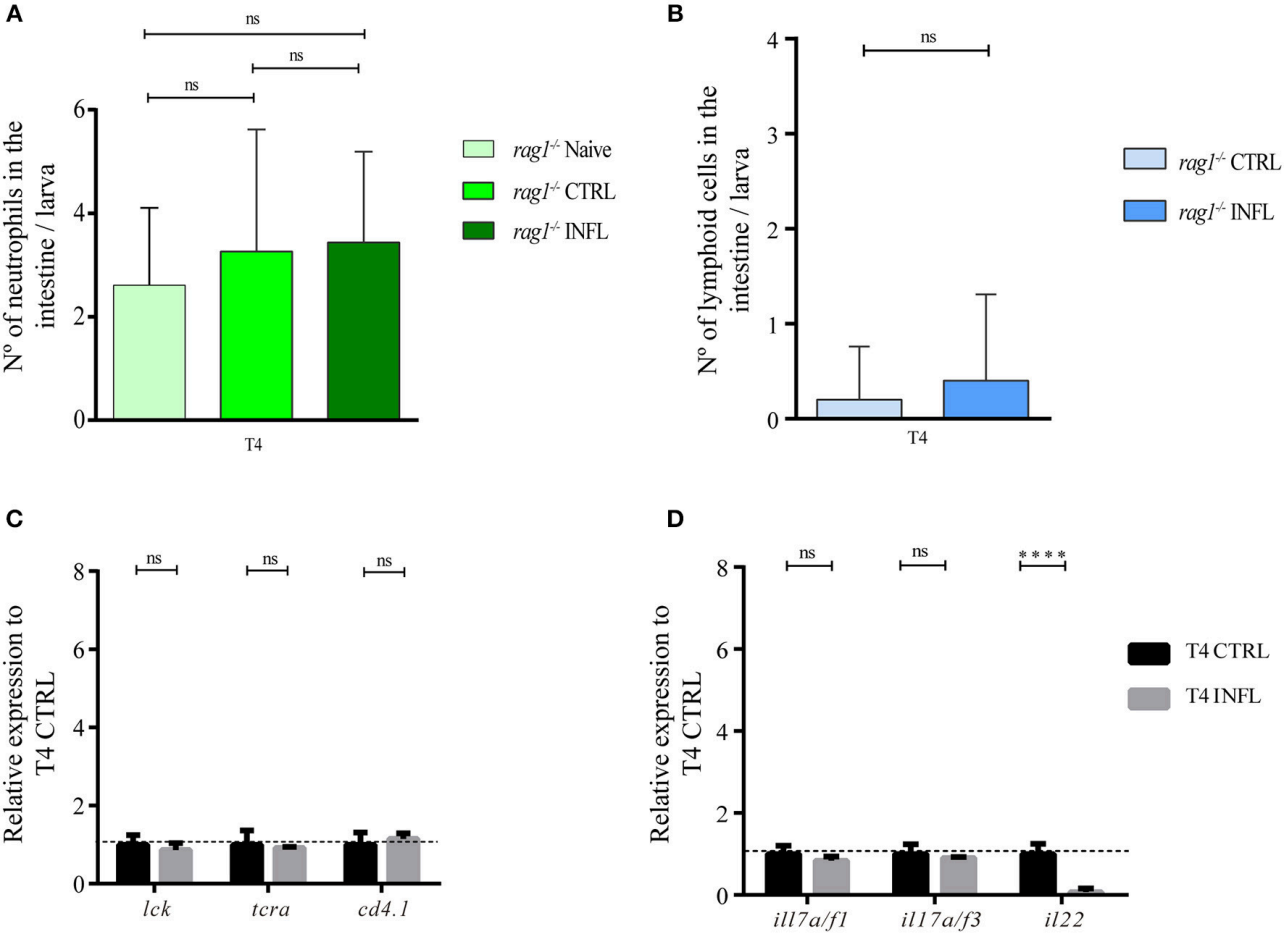
Soybean meal based inflammatory diet triggered intestinal inflammation in a T cell dependent manner. **(A)** Neutrophils quantification in naïve, control and inflamed *rag1*^−/−^ Tg(lck:lck-eGFP) larvae fed according to naïve model. **(B)** Lymphoid cells (lck^+^ cells) quantification in control and inflamed *rag1*^−/−^ Tg(lck:lck-eGFP) larvae fed according to naïve model. **(C–D)** Relative transcriptional level of lymphoid-associated genes were analyzed. Data was normalized against *rpl13a* and compared to the control condition (T4; dotted line). Neutrophils and lymphoid cells quantification were done at least in three biological replicates and with 20 individuals per condition. For each condition in RT-qPCR, 100 guts were analyzed, and three biological replicates were made. ^****^p > 0.0001.

To determine if the inflammation triggered by the intake of inflammatory diet was T cell-dependent, we carried out the naïve model in *rag1*^−/−^ larvae, which lack adaptive lymphocytes. First, we quantified number of neutrophils in intestines of larvae without feeding as well as in larvae fed with control and inflammatory diet. We found no significant difference between these three conditions; in naïve larvae, the average of neutrophils per intestine was 2.65 cells; in larvae fed with control diet was 2.95 cells and in larvae fed with the inflammatory diet was 3.2 cells (Figure 5A). Likewise, we performed the naïve model in *rag1*^−/−^ Tg(lck:lck-eGFP), and evaluated if the number of lck^+^ cells (innate lymphocytes and/or natural killer cells) increase in the intestine of larvae fed the inflammatory diet compared to control larvae. Our results showed a very low presence of lck^+^ cells in intestines in both conditions, with an average of 0.2 lck^+^ cells in larvae fed the control diet and 0.4 in larvae fed the inflammatory diet (Figure 5B). Regarding mRNA levels of other lymphoid markers, neither *lck, trac*, nor *cd4.1* increased expression level in intestines of larvae fed the inflammatory diet compared to those fed the control diet (Figure 5C). In the case of mRNA level of *mhc1zea* and *mhc2ab*, the first decrease their level and the latter increase them (Supplementary Figure 2). Likewise, the Th17 makers, *il17/f1* and *il17/f3*, did not change between the control and inflammatory conditions (Figure 5D). Altogether, these results indicate that the inflammatory process triggered by the intake of soybean meal-based diet is T cell-dependent.

## Discussion

The understanding of biological processes associated with intestinal inflammatory diseases such IBD has historically been a very active research focus due to the high prevalence of these pathologies worldwide. Most of these investigations are based on inflammation models developed in mice which, despite having allowed important advances, are not able to completely encompass the hallmarks of this disease. Thus, in this work we used an intestinal inflammation model established in zebrafish larvae based on the intake of soybean meal in which we monitored *in vivo* the participation of innate and adaptive cells. Specifically, we studied the recruitment of neutrophils, macrophages, mast cells, and T cells to the gut. We show here for the first time that, as early as 5 dpf, T cells are present in the intestine of zebrafish during homeostasis and that they increase in numbers upon soybean meal-induced inflammation. Thus, our results demonstrate that adaptive immune response is already functional at the end of the first week of zebrafish development.

In the case of myeloid cells, we observed a strong increase of macrophages numbers in the intestine in both models of inflammation. A similar phenomenon was observed for neutrophils. On the other hand, we did not detect an increase in the number of mast cells in any of the conditions studied. In humans, disorders such as IBD develop with a large infiltration of neutrophils, macrophages and monocytes to the gut (40, 41). Meanwhile, mast cells undergo activation, leading to a substantial release of mediators such as histamine and proteases (42). Surprisingly, we detected not only an increase in the number of macrophages present in the gut, but also drastic changes in their morphology between conditions where intestines have not yet faced food antigens compared to intestines that have experienced antigen encounter. In the first, all macrophages displayed a rounded shape and, in the latter, a combination of two morphologies was observed: rounded macrophages were accompanied by others with a clear increase in size and presence of long protrusions. Importantly, both macrophage types were observed in the control situation or under inflammatory conditions. Our RT-qPCR analysis indicated that in the control situation of the naïve model, the intestinal response was not tolerogenic but inflammatory. Thus, we speculate that these two morphologies could correspond to M0/homeostatic (round) and M1/pro-inflammatory (with protrusions) states. In mammals, different macrophage populations can be found. In a steady state gut, macrophages are characterized by very high levels of CX3CR1 expression, are avidly phagocytic and MHCII^hi^, but are resistant to Toll-like receptor stimulation, produce interleukin 10 constitutively, and express CD163 and CD206. Also, these cells have a round morphology (43). On the contrary, during an inflammatory process, macrophages express intermediate levels of CX3CR1, are Toll-like receptor responsive and pro-inflammatory, expressing IL6 and iNOS (43). Another possibility, based on the morphology of both macrophage populations in zebrafish larvae, is that the rounded cells are indeed monocyte and the cells with protrusions correspond to monocyte-derived dendritic cells (DCs). It has been shown that human CD16^+^ monocytes differentiate into migratory DCs during the inflammatory process (44). Likewise, and using CX3CR1 GFP^+^ mice in which monocytes and their daughter cells were tracked by analyzing GFP^+^ cells, Qu et al. (45) demonstrated that the Ly6C^+^ inflammatory monocyte subset gives rise to dendritic cells that migrate to lymph nodes and express Gr1.

Regarding lymphoid cells, we detected transcripts from a very early developmental stage (5 dpf) of *lck, trac*, and *cd4.1* genes, suggesting that the lymphoid cells observed in the intestine were helper T cells. Detection of *cd4.1* mRNA at early larval stages has been described before (46). Importantly, we and others (46) did not detect transcription of *cd8*, suggesting absence of Cd8^+^ T cells at this developmental stage. The *lck* gene has been recently shown to be expressed by zebrafish intestinal innate lymphoid cells (ILCs) (47). Within the ILC family, the ILC3 subset also expresses Th17 cytokines such as Il-22 and Il-17. Furthermore, mouse ILCs populate the intestine at earlier developmental stages than T cells, being crucial in inflammatory responses against enteric viruses in neonatal mice (48). We observed very low numbers of *rag1*^−/−^ *lck*-eGFP^+^ cells, thus ILCs, in the intestines of larvae in both control and soybean meal diet fed fish. In addition, we found lack of inflammation upon soybean meal diet in T-cell deficient fish (*rag1*^−/−^). Altogether, our data suggests that zebrafish ILCs do not play a role in soybean meal-induced inflammation. It remains to be determined the developmental stage at which ILCs populate the intestine of zebrafish as well as their participation in intestinal inflammation.

At the molecular level, it is interesting that in the control condition, the intestine displayed opposite responses in the two models used. In the case of the naïve model, the intestine faced food antigens for the first time, triggering an inflammation with increased expression levels of *il17a/f3*, and more importantly, without a tolerogenic response. Treg cell markers were unaltered and even decreased compared to the situation prior to feeding. Conversely, when the intestine had been previously exposed to food antigens, as in the developed model, we observed polarization of T cells toward a tolerogenic profile showing increased levels of *foxp3* and *il10* transcripts. These results suggest that the intestine must be educated to develop food tolerance even in the case of an innocuous antigen.

On the other hand, the inflammatory condition, both in the naïve and the developed models, elicited a clear Th17 response, with increased mRNA levels of the markers *il17a/f1, il17a/f3*, and *il22*, and decreased levels of the Treg markers *foxp3* and *il10*. Studies in mice and humans indicate that Th17 cells play a major role in the pathogenesis of Crohn's disease and ulcerative colitis (49–51). Furthermore, a correlation between disease severity and levels of IL-17 secreted by peripheral blood mononuclear cells from ulcerative colitis patients has been observed (52).

Regarding the signaling controlling T cell homing to the intestine during inflammation, our results suggest implication of Ccl25. As in mammals (53), we observed a considerable increase in the transcriptional level of *ccl25* in the intestine during inflammation. It remains to be determinate if Ccr9 is the cognate receptor of Ccl25 in zebrafish. Finally, we observed increased motility of lck-eGFP+ cells under inflammatory conditions, suggesting a functional change of these cells. The role of increased motility as well as the signals regulating this phenomenon remain to be explored. Importantly, *in vivo* imaging of intestinal lymphocytes upon different conditions, highlights the utility of the zebrafish model to analyze behavioral changes of different cell types *in situ* and in a non-invasively manner.

An important aspect we did not cover in this work is the participation of the microbiota in the induction of the soybean meal-induced intestinal inflammation. It is widely accepted is that IBD is triggered only under the presence of microbes, thus it remains to be determined whether our inflammation model is dependent on the presence of intestinal microbiota.

In summary, these two new intestinal inflammation models recapitulate many of the hallmarks of IBD observed in mice and humans, offering opposite situations that allow to generate a broad vision of the intestinal inflammatory process. This fact, added to the key advantages offered by the zebrafish model, positions our inflammatory model in a favorable position to offer a complementary alternative to the currently available IBD murine models.

## Ethics Statement

All animals were handled in strict accordance with good animal practice as defined by the European Union guidelines for the handling of laboratory animals and the Bioethics Committee of the Universidad Andres Bello, which approved this study, certificate number 007-2016.

## Author Contributions

MC, PH, and CF contributed to the conception and design of the study. MC and CS developed the experiments and performed the statistical analysis. CF wrote the first draft of the manuscript. All authors contributed to revising the manuscript, reading, and approving the submitted version.

### Conflict of Interest Statement

The authors declare that the research was conducted in the absence of any commercial or financial relationships that could be construed as a potential conflict of interest.

## Supplementary Material

Supplementary Figure 1 **(A)** Experimental strategy for naïve and developed feeding model. In the case of the naïve feeding model, 5 dpf larvae were fed with control diet (fishmeal-based diet) or inflammatory diet (soybean meal-based diet) during 4 days until 9 dpf. Samples were collected before and after feeding and behavior of neutrophils, macrophages, mast cells, and lymphocytes was analyzed. Also, transcriptional levels of different immune gene markers were evaluated. In the case of the developed feeding model, larvae were fed from 5 to 17 dpf with a commercial diet and then changed to control or inflammatory diet for 8 days (18–25 dpf). Before, at half time and after feeding, samples were collected and analyzed as for the naïve feeding model. **(B)** The amount of neutrophils was quantified in control and inflammatory conditions at T4 and at T4+4. **(C)** Transversal paraffin cross sections of the intestine of larvae fed according to the developed model stained with hematoxylin and eosin. **(D)** Quantification of the number of intestinal folds in control and inflamed larvae fed according the developed model. ^*^*p* < 0.05; ^***^*p* < 0.001.

Supplementary Figure 2 Relative transcriptional level of *mhc1zea, mhc2ab, hamp, leap2, trim33*, and *il23* genes were analyzed in wild type and *rag1*^−/−^ larvae after performed the naïve model. Data was normalized against *rpl13a* and compared to the control condition. For each condition, 100 guts were analyzed, and three biological replicates was made. ^**^*p* < 0.01; ^***^*p* < 0.001; ^****^*p* > 0.0001.

Supplementary Movie 1 Time lapse showing lck^+^ cells, lymphocytes, in the intestine of a Tg(lck:lck-eGFP) larva fed the control diet in the naïve model.

Supplementary Movie 2 Time lapse showing lck^+^ cells, lymphocytes, in the intestine of a Tg(lck:lck-eGFP) larva fed the inflammatory diet in the naïve model.
