# Supplementary figures and images for: Soybean Meal-Induced Intestinal Inflammation in Zebrafish Is T Cell-Dependent and Has a Th17 Cytokine Profile
Source: Front Immunol. 2019 Apr 2;10:610. doi: 10.3389/fimmu.2019.00610 (PMC6454071; doi:10.3389/fimmu.2019.00610)

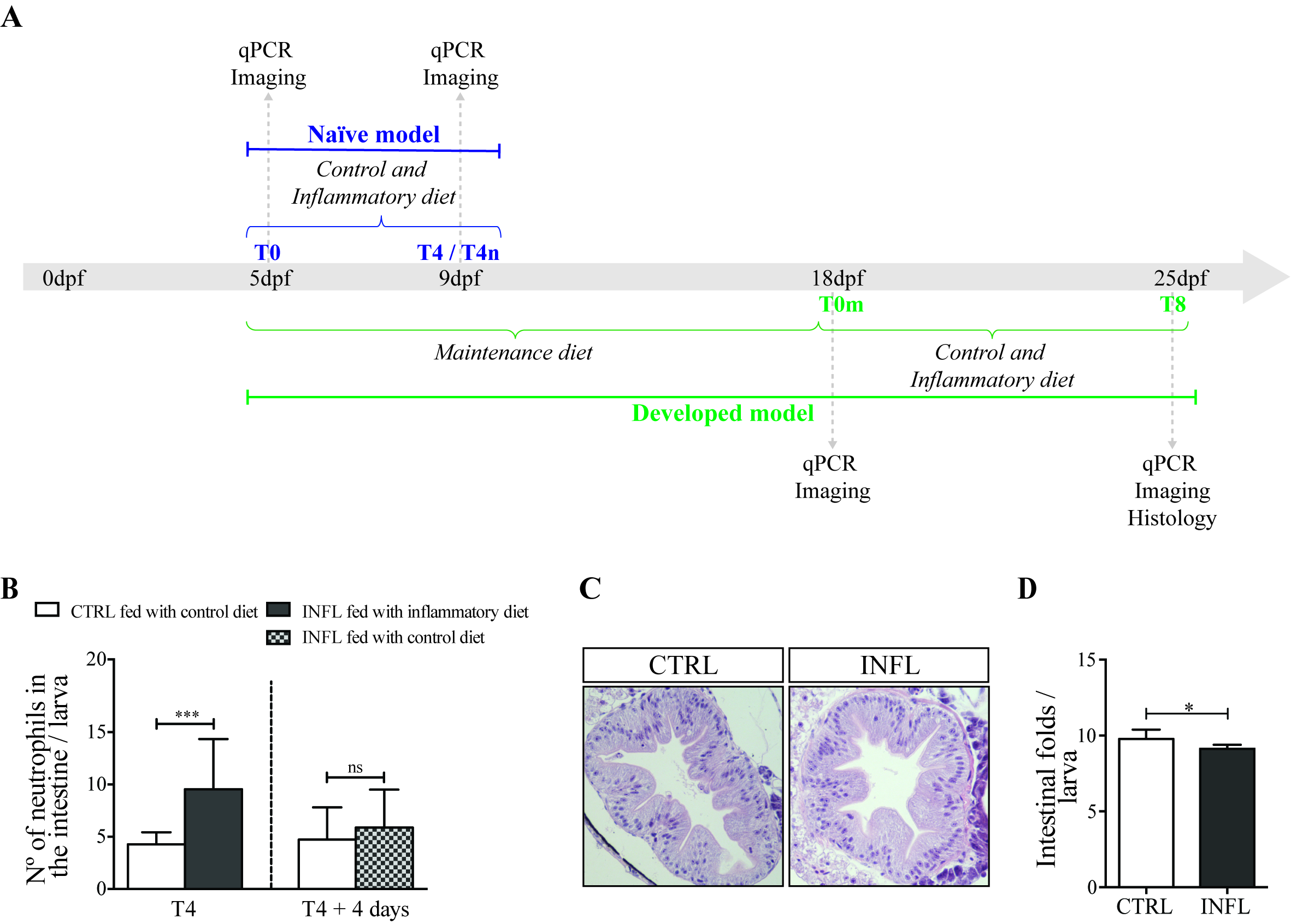

Supplement: Supplementary Figure 1 — (A) Experimental strategy for naïve and developed feeding model. In the case of the naïve feeding model, 5 dpf larvae were fed with control diet (fishmeal-based diet) or inflammatory diet (soybean meal-based diet) during 4 days until 9 dpf. Samples were collected before and after feeding and behavior of neutrophils, macrophages, mast cells, and lymphocytes was analyzed. Also, transcriptional levels of different immune gene markers were evaluated. In the case of the developed feeding model, larvae were fed from 5 to 17 dpf with a commercial diet and then changed to control or inflammatory diet for 8 days (18–25 dpf). Before, at half time and after feeding, samples were collected and analyzed as for the naïve feeding model. (B) The amount of neutrophils was quantified in control and inflammatory conditions at T4 and at T4+4. (C) Transversal paraffin cross sections of the intestine of larvae fed according to the developed model stained with hematoxylin and eosin. (D) Quantification of the number of intestinal folds in control and inflamed larvae fed according the developed model. *p < 0.05; ***p < 0.001. [file Image_1.TIF]

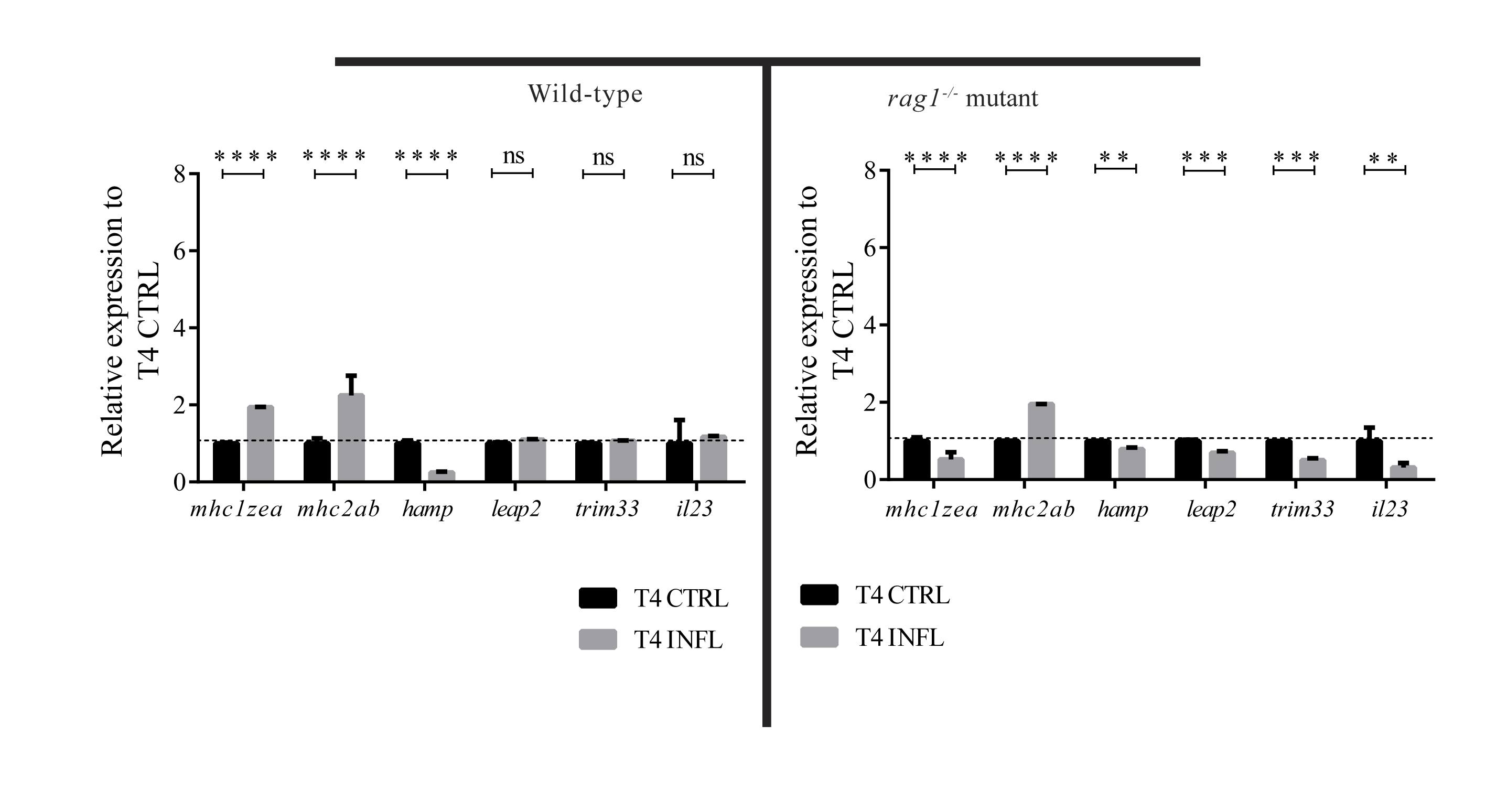

Supplement: Supplementary Figure 2 — Relative transcriptional level of mhc1zea, mhc2ab, hamp, leap2, trim33, and il23 genes were analyzed in wild type and rag1−/− larvae after performed the naïve model. Data was normalized against rpl13a and compared to the control condition. For each condition, 100 guts were analyzed, and three biological replicates was made. **p < 0.01; ***p < 0.001; ****p > 0.0001. [file Image_2.TIF]
